# Bullying victimization and associated factors among school-aged adolescents in Africa: a systematic review and meta-analysis

**DOI:** 10.1371/journal.pone.0321820

**Published:** 2025-04-24

**Authors:** Gidey Rtbey, Fantahun Andualem, Girum Nakie, Setegn Fentahun, Mamaru Melkam, Getasew Kibralew, Gebresilassie Tadesse, Belete Birhan, Techilo Tinsae, Girmaw Medfu Takelle

**Affiliations:** 1 Department of Psychiatry, College of Medicine and Health Sciences, University of Gondar, Gondar, Ethiopia; 2 College of Medicine and Health Sciences, Wolaita Sodo University, Wolaita Sodo, Ethiopia; Sadat Academy for Management Sciences, EGYPT

## Abstract

**Background:**

Bullying victimization during school age is a global public health concern. School-aged adolescents experiencing bullying victimization are more likely to encounter physical, cognitive, and mental health issues; including greater rates of depression, anxiety, and suicidal behaviors. This systematic review and meta-analysis was conducted to estimate the pooled prevalence of bullying victimization and its determinants among school-aged adolescents in Africa.

**Methods:**

All studies reporting the prevalence of bullying victimization and its determinants among African school-aged adolescents were included based on the predefined eligibility criteria. We followed the Preferred Reporting Items for Systematic Review and Meta-Analysis(PRISMA), a guideline for reporting a systematic review and meta-analysis. A random-effects model was employed to estimate the pooled effect size of bullying victimization and its determinants with their odds ratio (OR) and a 95% confidence interval(CI). Funnel plots analysis and Egger’s regression test were conducted to assess publication bias. Subgroup and sensitivity analyses were also performed.

**Results:**

Twenty-five studies involving 41,716 school-aged adolescents were included in this systematic review and meta-analysis. The overall pooled prevalence of bullying victimization among school-aged adolescents in Africa was 46.35%, with a 95% CI (41.45, 51.24). According to the subgroup analysis of the study region, 49.17% of bullying victimization was reported in the Eastern region of Africa whereas, 32.73% was reported in the Southern region. Engaging in physical fights [OR=1.86; 95% CI: 1.66, 2.07], current substance use [OR=1.85; 95% CI: 1.31, 2.62], feeling lonely [OR=1.98; 95% CI: 1.49, 2.65], and being worried [OR=2.56; 95% CI: 2.12, 3.1] were significantly associated with bullying victimization.

**Conclusion and recommendation:**

This review revealed that the pooled prevalence of bullying victimization among school-aged adolescents in Africa was high. To ensure adolescents’ mental health and cultivate productive manpower, fostering a supportive environment in schools is mandatory.

## Introduction

Bullying victimization is a frequent form of violence that children and adolescents experience at school ages [1,2]. Bullying victimization refers to either direct or indirect behavioral acts in which the perpetrator, who is typically powerful, intentionally abuses the victim verbally (insulting, teasing, or threatening), physically (coercion, hitting, pushing, kicking, or forcefully taking possession of someone’s belongings), or emotionally (social exclusion, spreading false information about someone) regularly [3–5].

The power imbalance between the perpetrator and the victim typically manifests as physical strength and status within the group (e.g., a group focusing on a single individual). It also takes advantage of a persons vulnerabilities (e.g., appearance, learning disability, family situation, or personal traits) and uses that information to hurt that person [1]. Beyond this, bullying in the modernized world can be committed using electronic devices and internet access to abuse the victim, referred to as cyber bullying [1,6–8].

The groundwork for future health and well-being is established in adolescence, making adolescence an important developmental stage [9]. The cognitive, emotional, sexual, and psychological growth of healthy adolescents is significantly enhanced by a supportive atmosphere of peer and parental interactions [10]. Bullying victimization during school age is a major public health concern, as adolescents who experience bullying are more likely to develop physical, cognitive, and mental health problems; including higher rates of depression, and anxiety [11,12].

Bullying is a prevalent problem in many nations and schools, affecting 20–56% of adolescents worldwide each year [13,14]. A global study using the Global School-based Student Health Survey(GSHS) of school children aged 12–17 years, between 2003 and 2015 revealed that the pooled prevalence of bullying victimization in one or more days in the last month was 30.5%, with the highest prevalence in the Eastern Mediterranean region (45.1%) [15]. Another study conducted in 66 countries using Global School Health Survey (GSHS) and Health Behavior in School-aged Children (HBSC) data of 2001/2 showed that the prevalence of bullying victimization was 37.4%(GSHS) and 32.1% (HBSC) [16].

Another study involving 40 countries using Health Behavior in School-aged Children data(2005/6) also indicated that 30% of adolescents reported experiencing bullying victimization in the past two months [17]. A comparative study conducted in low-and middle-income countries (LMICs) employing GSHS data collected between 2009 and 2015 showed that 34.4% of school adolescents experienced bullying victimization [18]. Bullying victimization is a widespread issue affecting school-aged adolescents in Africa, manifesting in various forms such as physical, verbal, and relational bullying [19]. In Africa, as a continent, different investigations were conducted to assess bullying victimization among school-aged adolescents ranging between 30.4% and 85.7% [20–22]. Bullying victimization was found to be lower in South Africa and Egypt [23,24], whereas the burden of the problem was higher in Nigeria and Kenya [22,25]. A study conducted in Sub-Saharan African countries utilizing GSHS data revealed that the prevalence of bullying victimization was 38.8% [14].

Bullying victimization can have consequences of internalizing problems such as low self-esteem [26,27], increased anxiety and depression [2,11], poor social skills [28,29], suicidal behaviors [30], and poor physical health [31]. Bullying victims also frequently miss class, perform poorly in school, avoid school-related activities, use drugs more frequently, eventually become truant, and could develop learning disabilities and attention-deficit/hyperactivity disorder (ADHD) [32–34]. In addition, bullying victimization could lead adolescents to use illicit drugs and later engage in criminal behaviors such as carrying a weapon [11,35]. Compared to those who experienced direct and relational bullying, adolescents who experienced cyber bullying were more likely to engage in self-harm and suicidal attempts. Females who experienced relational bullying were more likely than male adolescents to attempt suicide [36].

The available literature indicates that male sex, low socioeconomic status, younger age, and lower academic performance are positively associated with bullying victimization, whereas higher levels of peer and parental support are protective against bullying victimization [15,17,37]. Furthermore, adolescents with feelings of loneliness, a history of anxiety, or suicidal ideation and adolescents who use marijuana were more likely to be victims of bullying [14].

Despite the negative physical health and psychological implications of bullying victimization among school-aged adolescents, this issue has received less research attention in Africa. Although more studies have been conducted on bullying victimization in high-income countries, including systematic reviews, this study is the first to be conducted in Africa at the continental level to provide up-to-date evidence. Therefore, this review was conducted to estimate the pooled prevalence of bullying victimization and its determinants among African school-aged adolescents.

## Methods

### Study design, protocol registration

Systematic review and meta-analysis were conducted according to the Preferred Reporting Items for Systematic Review and Meta-Analysis (PRISMA 2020) standard [38] (S1 File). This study’s protocol was registered in the International Prospective Register of Systemic Review (PROSPERO) (ID= CRD42024550067).

### Search strategies

The search strategy was intended to explore all available literature addressing the prevalence of bullying victimization and associated factors among school-aged adolescents in Africa. Initially, a search on databases like PubMed/ Medline, Science Direct, EMBASE, and African Journals Online (AJOL) was performed followed by checking the text words contained in title/abstract and indexed terms. Secondly, a search that combined free text words and indexed terms with Boolean operators was conducted. The search method for each database was performed with different Boolean operators for each database accordingly (S2 File). The third search was performed with the reference lists of all identified reports and articles for additional studies.

Furthermore, a search for any other gray literature was attempted by searching Google and Google Scholar to identify any additional articles through the end of April 2024. The search terms used were “bullying victimization” and associated factors among school adolescents or other medical subject heaving (MeSH), keywords, and free text search terms. The authors used several phrases, such as bullying victimization OR cyber bullying victimization AND school adolescents, and combined terms by utilizing Boolean operators while searching for the literature. The search terms used were (“bullying victimization OR cyber bullying victimization”) AND (“associated factors” OR “determinants” AND “school adolescents” OR “high school students” OR “school going adolescents”) AND “Africa (inserting each African country”).

### Eligibility criteria

In accordance with the Joanna Briggs Institute’s recommendation [39], the inclusion criteria were developed using the mnemonic “Coco Pop” (Table 1). Coco Pop comprises of: Condition (the health condition, disease, symptom, event, or factor); Context (the environmental factors that impact the prevalence or incidence of the condition, such as the geographical area, and the setting); and Population (an outline of the defining population characteristics). All peer-reviewed journal articles and gray literature that addressed bullying victimization and/or associated factors among school-aged adolescents and that were written in the English language were selected for this systematic review and meta-analysis. All cross-sectional studies reporting the prevalence of bullying victimization and its determinants studies conducted among school-aged adolescents in any African country were included in this review. Studies that lacked an abstract, did not have a full text and for which it was difficult to obtain the necessary data after contacting their respective authors were excluded from this systematic review and meta-analysis.

**Table 1:** Eligibility criteria for screening studies applying the CoCoPop (condition, context, and population) protocol.

Categories	Inclusion	Exclusion
Condition	Bullying victimization among school-aged adolescents and the associated factors.	Studies not focused on bullying victimization or those focusing on bullying perpetration.
Context	Studies conducted in school settings in Africa, including both urban and rural contexts.	Research outside school settings or outside of Africa.
Population	School-aged adolescents (ages 10–19) in Africa attending school.	Studies on children not of school age or adolescents not attending school.

### Outcome measurement

In this study, the definition of our outcome variable (bullying victimization) was based on the assessment instruments designed to measure bullying victimization in school-aged adolescents. The included studies measured bullying victimization with a self-report question “During the past 30 days, how many days were you bullied?” GSHS and adolescents who experienced bullying once and above within the last month were considered to have bullying victimization. Some studies also employed the Olweus Bully/Victim Questionnaire(BVQ) to assess bullying victimization and according to this assessment tool, the cut-off point for classifying an adolescent as a victim or non-victim was defined as “2 or 3 times a month [40].

School Life Survey was also used to detect the frequency of bullying and victimization and four acts during the past month were the cut-off for defining bullying victimization [41]. The experience of bullying victimization was assessed using questions adapted from the Multidimensional Peer Victimization scale [42]. Adolescent Peer Relations Instrument (APR) which is an 18-item scale comprising 3 subscales on verbal, social, and physical bullying, was used to collect data on bullying victimization [43]. Forms of Bullying Scale (FBS), a self-report measure of adolescents’ exposure to bullying behavior and victimization version was used to assess bullying victimization. The second objective of this study was to determine factors associated with bullying victimization and factors were measured with measure of associations like AOR and β values. For the independent variables, the AOR with their 95% CI were taken to calculate the pooled effect size of the variables.

### Data extraction

All available studies from research databases and other additional searches were imported to the citation manager and duplication removal was conducted using the software. After the titles, abstracts, and full texts of the articles were thoroughly reviewed, the pertinent data were extracted using a Microsoft Excel Spreadsheet. The data were extracted using the authors names, years of publication, study design, study region, and sample size from the included articles. Additionally, the estimated combined effects of Bullying victimization and its associated factors were retrieved with 95% confidence intervals. GR and GN performed the primary data extraction and the arguments and differences were resolved through discussion with the fourth author GM (S3 File).

### Quality appraisal

Two authors (MM and SF) independently assessed the quality of the studies included in this systematic review and meta-analysis. The Joanna Briggs Institute (JBI) assessment tool was used to assess the quality of the studies [39]. The JBI assessment tool is commonly used in systematic reviews and research to evaluate the quality and methodological rigor of studies. The tool has nine items assessing the study design, sampling and data collection methods, data analysis, results, and conclusions of the primary studies. The quality assessment of selected articles was the same for all because all studies were cross-sectional. Articles with a final rating scale of five or more out of nine points were included in this study. Differences between reviewing authors in terms of quality ratings were resolved by discussion with another third author (GT) to reach a consensus (S4 File).

### Data analysis

The extracted data were exported to STATA version 14 statistical software for analysis. A random-effects model was employed to obtain the estimated pooled effect size and the effect size of all articles with a 95% confidence interval [44]. Heterogeneity was assessed using Cochran’s Q, the I**²** statistics, and p-values. The percentage of a study estimates overall variance that may be attributed to heterogeneity was measured using I^**2**^ statistics; 25%, 50%, and 75% indicated low, medium, and high heterogeneity respectively [45]. To declare study as having significant heterogeneity the p-value should be ≤ 0.05. Measures of associations (ORs and β values) were presented using narrative synthesis for the second objective, which involved identifying factors associated with bullying victimization. Adjusted estimates were calculated to interpret the associations between significant factors and bullying victimization. For the graphical summary, a forest plot was used to estimate the pooled effect size and weight of each recruited study with a 95% CI.

Publication bias was checked using a funnel plot and Egger’s weighted regression test. A p-value of <0.05 according to Egger’s test was considered to indicate statistically significant publication bias [46,47]. Subgroup analysis employing the year of publication and study region (Eastern, Western, Northern, and Southern Africa) was performed. A sensitivity analysis was conducted by omitting one study at a time to identify how each selected study affected the overall pooled prevalence of bullying victimization in this systematic review and meta-analysis. Moreover, meta-regression was done to point out the possible sources of heterogeneity. Since there was no missing data in the included studies we didn't conduct any missing data handling procedure other than sensitivity analysis.

## Results

### Study selection process

**Fig 1:**
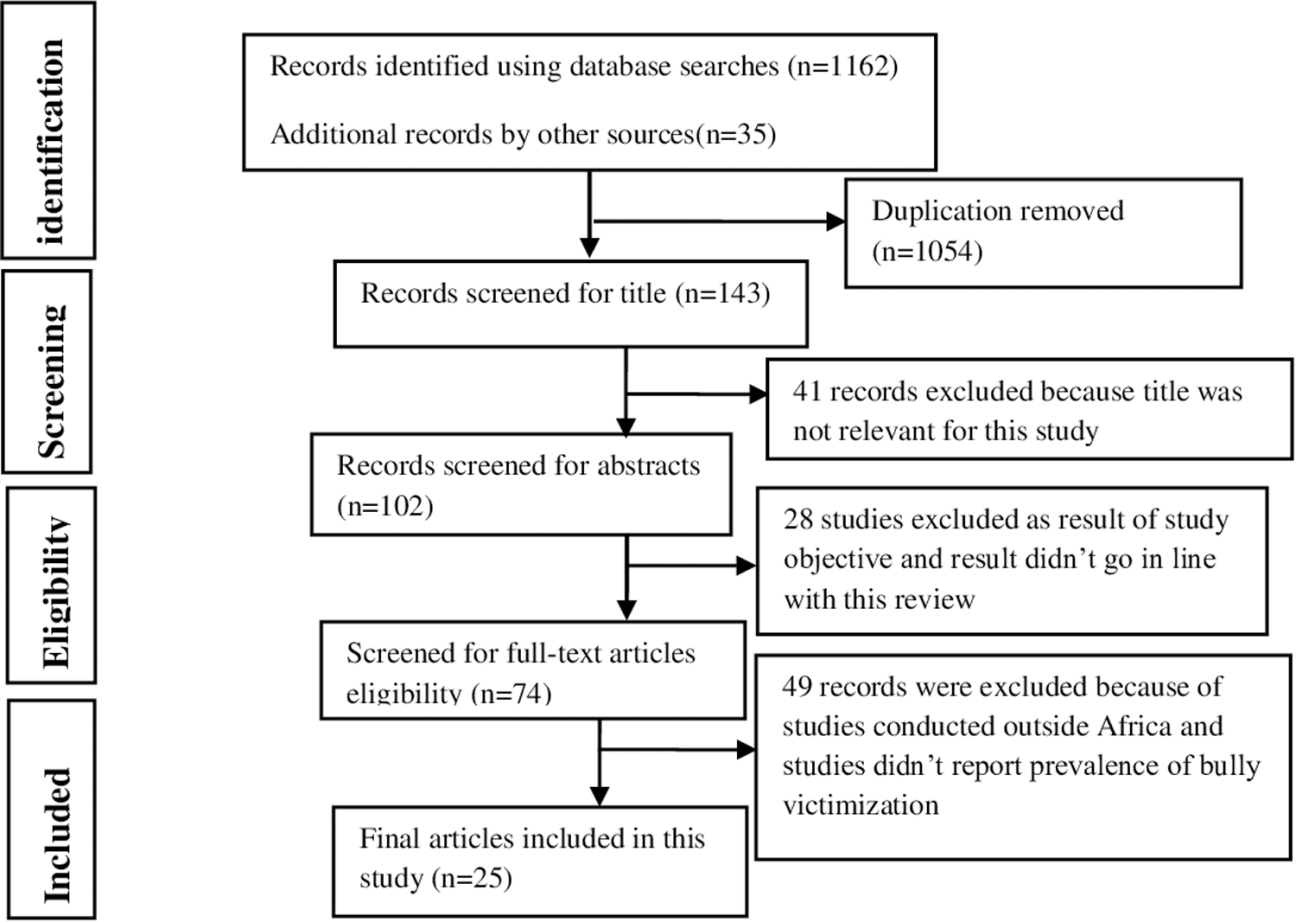
PRISMA flow chart describing the selection of primary studies in this systematic and meta-analysis.

Initially, 1162 studies were identified using different database searches, and 35 more studies were identified with additional searches (Google and Google Scholar). Then, due to duplication, 1054 studies were removed, and 41 papers were excluded because their titles and objectives were unrelated to this systematic review and meta-analysis. Of the 74 studies that passed through full-text screening, only 25 met the necessary criteria for this review (Fig 1).

### Description of included studies

In this systematic review and meta-analysis, a total of 25 primary studies on bullying victimization among school-aged adolescents in Africa were included. These included studies were conducted in Egypt (Two), Ethiopia (Three), Ghana(Three), Nigeria(Four), Tunisia(Two), and Algeria, Benin, Botswana, Eswatini(Swaziland), Kenya, Liberia, Malawi, Mozambique, Sierra Leone, South Africa, and Zambia one study in each country (Table 2).

**Table 2:** Characteristics of the studies included in this systematic review and meta-analysis on bullying victimization among school-aged adolescents in Africa.

Author’s name and year of publication	Study country	Age range in years	Sample size	Method of data collection	Prevalence of bullying victimization (%)
Aboagye et al., 2021 [65].	Ghana	12–18	1342	SA	41.3
Adeosun al., 2015 [66].	Nigeria	NR	380	SA	56.8
Fobi et al., 2022 [67].	Ghana	13-24	450	SA	55.1
Fredj et al., 2023 [68].	Tunisia	NR	791	SA	43.4
Galal et al., 2019 [69].	Egypt	12–18	476	IA	57.8
Ghardallou et al., 2024 [70].	Tunisia	11-17	1111	SA	45.8
Hirpa et al., 2018 [71].	Ethiopia	11-18	809	SA	37.6
Ighaede-Edwards et al., 2023 [72].	Nigeria	NR	621	SA	51.9
Khairy et al., 2021 [24].	Egypt	11–17	400	IA	29.8
Kubwalo et al., 2013 [73].	Malawi	13-15	2,264	SA	44.5
Mazaba-Liwewe et al., 2015 [21].	Benin	NR	2,690	SA	40.1
Mlisa et al., 2008 [23].	South Africa	NR	1311	SA	16.49
Mokaya et al., 2022 [22].	Kenya	11-18	539	SA	85.7
Okobi et al., 2023 [74].	Liberia	11-18	2,744	SA	50
Olumide et al., 2016 [75].	Nigeria	NR	653	IAEQ	39.8
Osborne et al., 2023 [76].	Sierra Leone	10–19	2,798	SA	48.7
Owusu et al., 2011 [77].	Ghana	NR	7,137	SA	40.1
Peltzer and Pengpid, 2020 [78].	Mozambique	NR	1918	SA	45.5
Raji et al., 2019 [25].	Nigeria	10-19	450	IA	65.6
Rudatskira et al., 2014 [79].	Algeria	NR	4532	SA	51.1
Sandhu et al., 2018 [80].	Ethiopia	11-18	809	SA	37.6
Shongwe et al., 2021 [20].	Eswatini	13–17	2920	SA	30.4
Siziya et al., 2012 [81].	Zambia	NR	1559	SA	62.8
Sricharan Pasupulati et al., 2013 [82].	Botswana	NR	2,165	SA	51.3
Tarafa et al., 2022 [83].	Ethiopia	10-19	847	IA	30.4

Note: NR: Not Reported, SA: Self-administered, IA: Interviewer administered, IAEQ: Interviewer-Assisted Electronic Questionnaire.

### Meta-analysis

**Fig 2:**
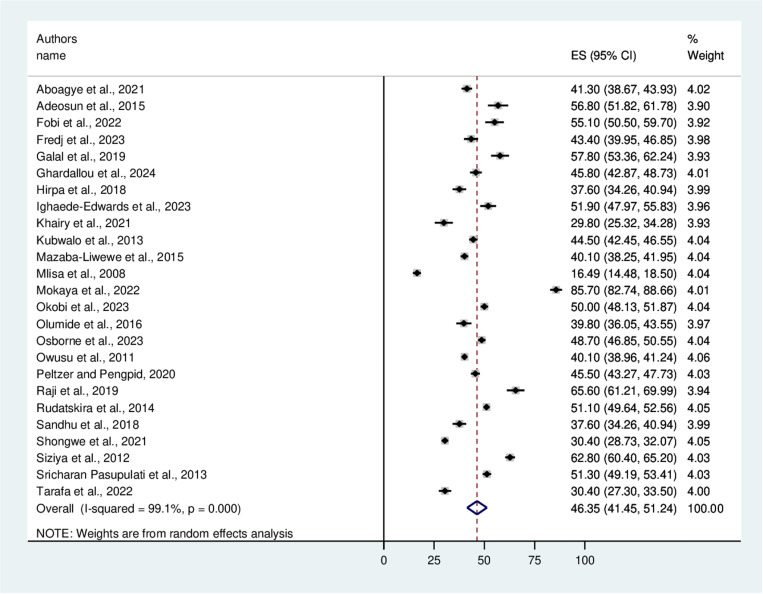
A forest plot showing the pooled prevalence of bullying victimization among school-aged adolescents in Africa.

This meta-analysis revealed that the overall pooled prevalence of bullying victimization among school-aged adolescents in Africa was 46.35% with a 95% CI (41.45, 51.24) (Fig 2). The results indicated that there was considerable heterogeneity (I^**2**^ =99.1%). Using the univariate meta-regression model, several heterogeneity-related parameters, including sample size (p=0.99) and year of publication (P=0.92), were investigated in an attempt to pinpoint the potential source of heterogeneity. However, none of these variables were determined to be statistically significant.

### Heterogeneity and publication bias

**Fig 3:**
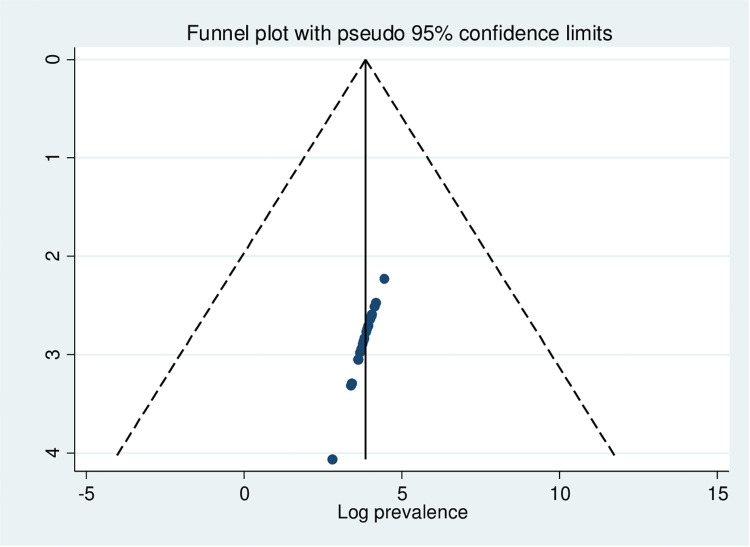
Funnel plots of bullying victimization among school-aged adolescents in Africa.

In this systematic review and meta-analysis, heterogeneity was detected, with an I^**2**^ of 99.1% and a P-value of 0.001. Funnel plots were used to assess publication bias, and the absence of publication bias was confirmed by inspection of the seemingly symmetrical distribution of the plots (Fig 3). Furthermore, Egger’s regression test was also employed and confirmed that there was no publication bias (P= 0.29) (Table 3).

**Table 3:** Egger’s tests of bullying victimization among school-aged adolescents in Africa.

Std_Eff	Coef.	Std. Err.	t	P>t	[95% Conf.	Interval]
slope	37.6659	6.379947	5.90	0.000	24.46798	50.86383
bias	5.86444	5.409846	1.08	0.29	-5.32668	17.05556

### Subgroup analysis

**Fig 4:**
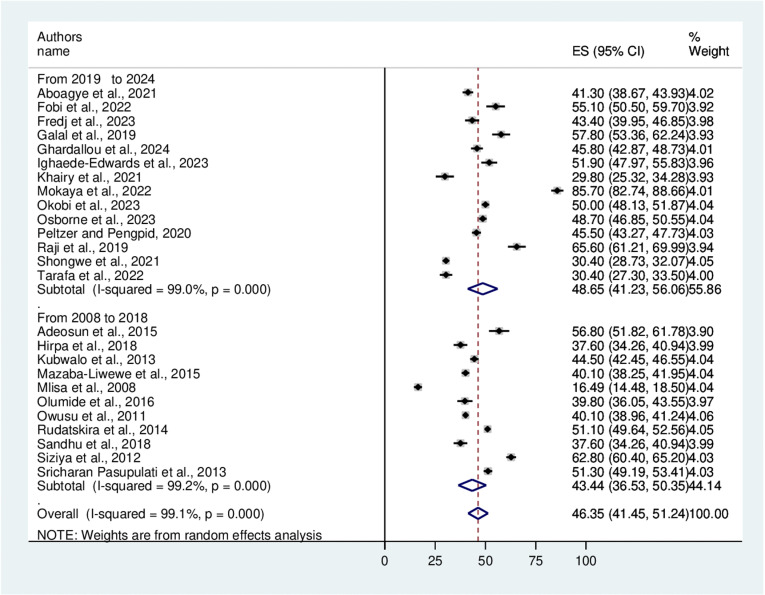
Forest plot showing a subgroup analysis based on the year of publication.

**Fig 5:**
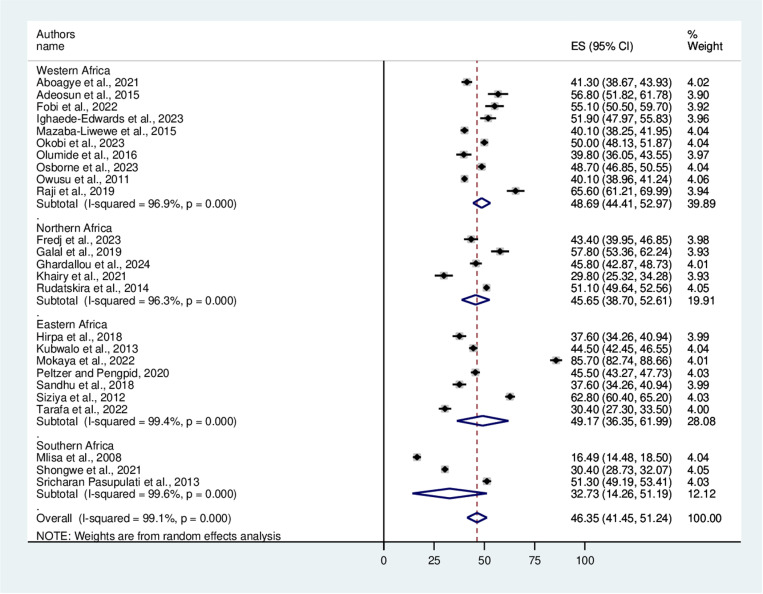
Forest plot showing a subgroup analysis based on the study region.

After confirming the presence of heterogeneity, a subgroup analysis based on the year of publication and study region was conducted. According to the subgroup analysis of year of publication, bullying victimization accounted for 43.44% and 48.65% of the studies published from 2008 to 2018 and from 2019 to 2024, respectively (Fig 4). Moreover, the study region revealed that bullying victimization was 48.69% in Western Africa, 45.65% in Northern Africa, 49.17% in Eastern Africa, and 32.73% in the Southern part of the continent (Fig 5).

### Sensitivity analysis

**Fig 6:**
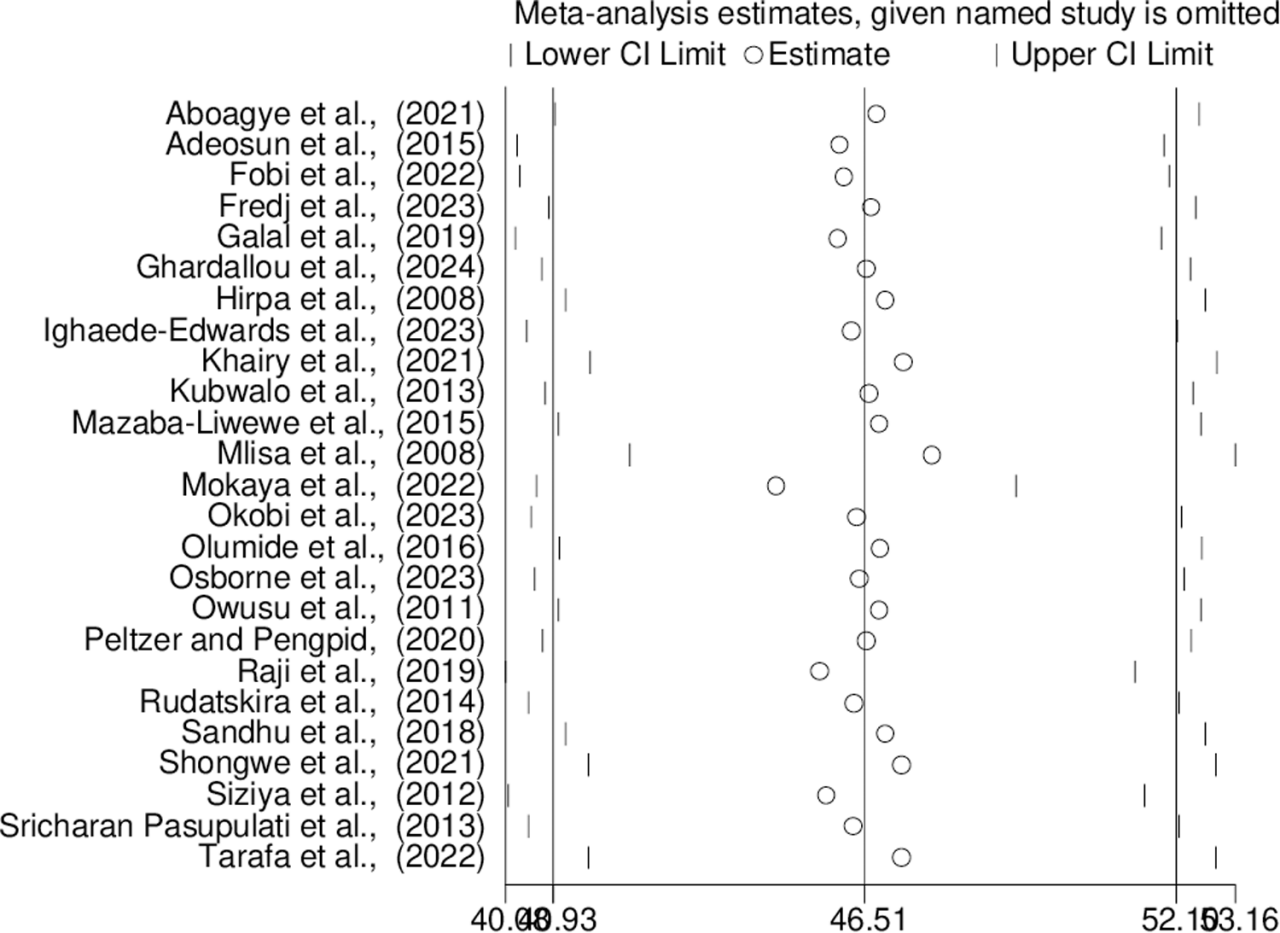
Sensitivity analysis for studies included in this systematic review and meta-analysis.

By excluding one study at a time, a sensitivity analysis was conducted to determine how each selected study affected the overall pooled prevalence of bullying victimization in this systematic review and meta-analysis. The sensitivity analysis revealed that all values were almost within the estimated 95% CI, indicating that the results of this meta-analysis were not significantly affected by the omission of a single study (Fig 6).

### Determinants of bullying victimization

In this systematic review and meta-analysis, several factors contributing to bullying victimization among adolescents were identified. School adolescents who engaged in physical fights were 1.86 times more likely to experience bullying victimization than their counterparts [OR=1.86 (1.66, 2.07)]. Compared to adolescents who did not use any substance, adolescents who used substances were at higher risk of experiencing bullying victimization [OR=1.85 (1.31, 2.62)].

**Fig 7:**
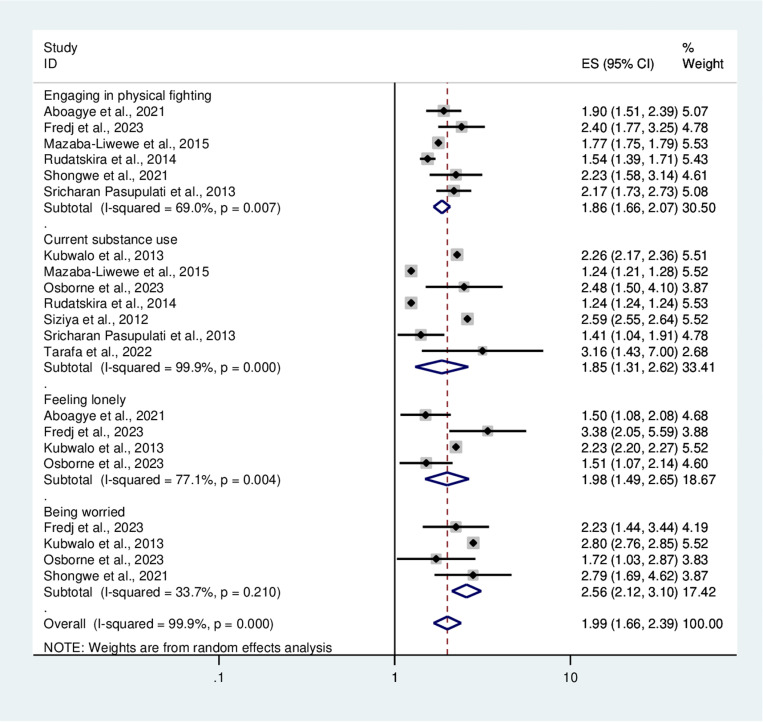
Forest plot showing factors associated with bullying victimization.

Participants who experienced feelings of loneliness were approximately 2 times more likely to become bully-victims than their counterparts [OR=1.98 (1.49, 2.65)]. Adolescents who were worrying about many things were more likely to be bullied than those who did not worry [OR=2.56 (2.12, 3.1)] (Fig 7).

## Discussion

Bullying victimization is one of the challenges adolescents face in their school stays. It has a detrimental effect on academic performance, psychological and emotional health of school-adolescents. To estimate the burden of the problem at a continental level, this systematic review and meta-analysis included 25 primary studies conducted on bullying victimization and associated factors among school-aged adolescents in Africa involving a total of 41,716 adolescents.

This study revealed that the overall pooled prevalence of bullying victimization among school-aged adolescents in Africa was 46.35%, with a 95% CI (41.45, 51.24). This finding was consistent with a study in Nepal (51%) [2]. However, this finding was higher than the study conducted among 66 countries using GSHS data (37.4%) [16], a study that utilized Health Behavior in School-aged Children (HBSC) data (30%) [17], and low- and middle-income countries (34.4%) [18]. This finding was also lower than studies in the Solomon Islands (65.7%), Vanuatu (67.9%), and Samoa (74.2%) [48]. The possible reasons for the variations in the prevalence of bullying victimization might be due to the differences in the assessment tools used, school environment, parental support, and availability of anti-bullying supporting groups. Furthermore, socio-cultural, and economic variations and previous interventions to address the problem might have contributed to this inconsistency. Different countries and regions may have varying social dynamics, cultural norms, and attitudes toward bullying, which can influence how bullying is reported and perceived.

Subgroup analysis revealed that the eastern and western African regions had a higher prevalence of bullying victimization than did the southern part of the continent. This inconsistency might be due to differences in assessment tools, school environment, and sample sizes of the included primary studies. Though further region-specific research might be needed to reveal the differences, the availability of resources and teacher training across regions can affect how bullying is addressed. In areas like Northern Africa, where educational systems are often better funded or have more access to anti-bullying programs, bullying rates may be lower whereas, in regions like Western and Eastern Africa, where educational systems might face more challenges bullying could be less effectively addressed.

The burden of the problem was slightly higher in articles published between 2019 and 2024 than in studies published between 2008 and 2018, according to the subgroup analysis employing the year of publication. The emergence of new technologies, adolescent exposure to social media, changes in educational dynamics, and accessibility to electronic devices used for bullying could be contributing factors to the increment of the problem. Additionally, the growing emphasis on the effects of bullying on school activities and the mental health of school-aged adolescents may have contributed to a rise in research, resulting in higher reported prevalence rates.

Regarding the determinants of bullying victimization, adolescents who engaged in physical fights were more likely to be bully-victimized than their counterparts. This finding was supported by studies in Thailand [49] and Western Pacific Island Countries [50]. This could be explained by the fact that adolescents who are frequently bullied might be more prone to antisocial behavior and physical fights compared to their counterparts [11,51]. Moreover, a study showed that externalizing behaviors (aggression and delinquency) are both antecedents and consequences of bullying victimization [52].

School adolescents who were using substances like alcohol, cigarettes, or marijuana were more likely to be bully-victims than those who were not substance users. This result was corroborated by studies in Iraq [53], LMICs [54], the USA [55], and China [56]. Adolescent substance use and abuse can have a direct connection to bullying victimization of all types, according to a growing national and international study [57]. Although the relationship between bullying victimization and substance use is unclear and further investigations are needed to identify the temporal relationship between the two variables, there are controversies regarding this evidence; some researchers have claimed that externalizing behaviors (aggression and substance use) could lead adolescents to bullying victimization [52,58], while others stated that substance use is the adverse consequence of bullying victimization [57,59].

This systematic review and meta-analysis showed that the likelihood of being bullied was higher among school adolescents who felt lonely than their counterparts. This finding was consistent with studies in Spain [60], Nepal [2], and Denmark [61]. The reason for this may be as a result of being alone and not interacting with friends at school or outside school could lead adolescents to be bullied. Furthermore, lonely adolescents appear to be less accepted and more rejected by their friends [62,63], which makes those individuals vulnerable to being bullied by their peers compared to those with good social interactions [64].

The other variable associated with bullying victimization was being worried or having anxiety. Participants who experienced worries about different things were at increased risk of being bullied. This evidence was in line with previous studies [49,54]. Adolescents who have worries or experience anxiety symptoms may be embarrassed and bullied by misconceiving their speech and activity.

### Strengths and limitations of the study

This review used a comprehensive search to avoid missing available articles. The current study was conducted using the updated PRISMA 2020 guideline and utilized quality appraisal and data extraction by two investigators representing the main strength of this review. The absence of significant publication bias increases the reliability of the findings. However, all the primary studies included in this review were cross-sectional studies, which does not establish a real cause-and-effect relationship between the covariates and outcome variables. There was significant heterogeneity in both the overall meta-analysis and subgroup analysis. Furthermore, this review included studies from less than half of African countries, which might make it difficult to generalize at the continental level based on the current study’s findings.

### Implications of the study

It is known that the adolescent age is a crucial stage of an individual’s life and repeated experience of bullying victimization has detrimental effects on their later life. Addressing school bullying is a global priority and mandatory in achieving the Sustainable Development Goals (SDGs), in particular, SDG 4.a.2, to access safe, non-violent, inclusive, and effective learning environments for all, and SDG 16.2, to end all forms of violence against children, including bullying. Therefore, this study will be an input for policymakers and responsible officials. Furthermore, both governmental and non-governmental organizations can implement the findings of this study in developing anti-bullying supporting groups and other strategies in schools to tackle the problem. The findings may also serve as baseline information for future researchers to conduct longitudinal studies to further investigate the temporal relationship of the covariate and outcome variable.

## Conclusion and recommendation

This systematic review and meta-analysis indicated that the prevalence of bullying victimization among school-aged adolescents in Africa was high. Engaging in physical fights, current substance use, feeling loneliness, and being worried were factors significantly associated with bullying victimization in this review. Parents and school officials should be aware of the impacts of bullying among school adolescents and take part in interventions to address the identified factors associated with bullying victimization to reduce the burden of the problem. Create safe, inclusive school environments where every student feels secure, regardless of background. Focus on conflict resolution, anger management, and promoting positive behavior to reduce aggression and victimization. Implement substance use prevention programs and offer counseling to teach healthier coping strategies. Foster social inclusion, friendship-building, and peer support to reduce loneliness and strengthen school-community ties. Provide accessible mental health services to support students facing anxiety or emotional challenges.

Encouraging collaborative approaches between schools, local communities, governments, and non-governmental organizations (NGOs) can lead to more comprehensive and sustained efforts to address bullying. Conduct region-specific research to understand local variations in bullying, considering factors like urban vs. rural settings, and socio-economic influences. Furthermore, conducting a longitudinal study is needed to identify the cause-effect relationship between the outcome and covariate variables.

## Supporting information

S1 File PRISMA 2020 checklist for bully victimization. (DOCX)

S2 File Searching strategy. (DOCX)

S3 File Extracted data for bully victimization. (XLSX)

S4 File JBI quality assessment for bully victimization. (DOCX)

## Abbreviations

AJOL: African Journals Online
CI: Confidence Interval
GSHS: Global Students Health Survey
HBSC: Health Behavior in School-aged Children
LMICs: Low- and Middle-Income Countries
OR: Odds Ratio
PRISMA: Preferred Reporting Items for Systematic Review and Meta-Analysis
SNNPs: South Nationality and Peoples
USA: United States of America
WHO: World Health Organization.
