# Supplementary material for: Bullying victimization and associated factors among school-aged adolescents in Africa: a systematic review and meta-analysis
Source: PLoS One. 2025 Apr 24;20(4):e0321820. doi: 10.1371/journal.pone.0321820 (PMC12021201; doi:10.1371/journal.pone.0321820)
Supplement: S2 File — (DOCX) [file pone.0321820.s002.docx]

**Search strategy for research databases**

1. **PubMed/Medline**

(((((((((prevalence) OR (burden)) AND (bullying victimization)) OR (cyberbullying victimization)) AND (associated factors)) OR (determinants)) AND (school-aged adolescents)) or (high school students)) AND (Africa)) OR (African countries)

1. **Science Direct**

("Bullying victimization" OR "Bullying prevalence" OR "Cyberbullying") AND ''Associated factors'' OR ''determinants'' AND ("school-aged adolescents" OR "adolescents") AND ("Africa" OR "African countries")

1. **EMBASE**

(((('prevalence'/exp OR 'prevalence' OR 'epidemiology'/exp OR 'epidemiology' OR 'magnitude'/exp OR 'magnitude') AND (bullying victimization'/exp OR 'bullying victimization') OR 'cyberbullying'/exp OR 'cyberbullying') AND 'associated factors' OR 'risk factors'/exp OR 'risk factors') AND 'school-aged adolescents' OR 'school adolescents') AND ('Africa/exp OR 'Africa' OR 'each African country') AND [embase]/Lim

1. **African Journals Online (AJOL)**

("Bullying victimization" OR "Bullying prevalence" OR "Cyberbullying") AND ("school-aged adolescents" OR "adolescents") AND ("Africa" OR "African countries")
