# Supplementary material for: Bullying victimization and associated factors among school-aged adolescents in Africa: a systematic review and meta-analysis
Source: PLoS One. 2025 Apr 24;20(4):e0321820. doi: 10.1371/journal.pone.0321820 (PMC12021201; doi:10.1371/journal.pone.0321820)
Supplement: S4 File — (DOCX) [file pone.0321820.s004.docx]

**Table S1: JBI Quality assessments of selected studies in this systematic review and meta-analysis on bully victimization and associated factors among school adolescents in Africa.**

| **Author’s name and year of**  **Publication** | **Q1** | **Q2** | **Q3** | **Q4** | **Q5** | **Q6** | **Q7** | **Q8** | **Q9** | **Total score (9)** |
| --- | --- | --- | --- | --- | --- | --- | --- | --- | --- | --- |
| 1. Aboagye et al., 2021 | Y | Y | NA | Y | Y | Y | Y | Y | Y | 8 |
| 1. Adeosun et al., 2015 | Y | Y | Y | NR | Y | Y | Y | Y | Y | 8 |
| 1. Fobi et al., 2022 | Y | Y | Y | Y | Y | Y | Y | Y | Y | 9 |
| 1. Fredj et al., 2023 | Y | Y | Y | Y | Y | Y | Y | Y | Y | 9 |
| 1. Galal et al., 2019 | Y | Y | Y | NR | Y | Y | Y | Y | Y | 8 |
| 1. Ghardallou et al., 2024 | Y | Y | Y | Y | Y | Y | Y | Y | Y | 9 |
| 1. Hirpa et al., 2018 | Y | Y | NA | Y | Y | Y | Y | Y | Y | 8 |
| 1. Ighaede-Edwards et al., 2023 | Y | Y | NA | Y | Y | Y | Y | Y | Y | 8 |
| 1. Khairy et al., 2021 | Y | Y | NA | Y | Y | Y | Y | Y | Y | 8 |
| 1. Kubwalo et al., 2013 | Y | Y | Y | Y | Y | Y | Y | Y | NR | 8 |
| 1. Mazaba-Liwewe et al., 2015 | Y | Y | Y | Y | Y | Y | Y | Y | Y | 9 |
| 1. Mlisa et al., 2008 | Y | Y | NA | Y | Y | Y | Y | Y | Y | 8 |
| 1. Mokaya et al., 2022 | Y | Y | Y | NR | Y | Y | Y | Y | Y | 8 |
| 1. Okobi et al., 2023 | Y | Y | Y | Y | Y | Y | Y | Y | Y | 9 |
| 1. Olumide et al., 2016 | Y | Y | Y | NA | Y | Y | Y | Y | Y | 8 |
| 1. Osborne et al., 2023 | Y | Y | Y | Y | Y | Y | Y | Y | Y | 9 |
| 1. Owusu et al., 2011 | Y | Y | NA | NR | Y | Y | Y | Y | Y | 7 |
| 1. Peltzer and Pengpid, 2020 | Y | Y | Y | NA | Y | Y | Y | Y | Y | 8 |
| 1. Raji et al., 2019 | Y | Y | Y | Y | Y | Y | Y | Y | Y | 9 |
| 1. Rudatskira et al., 2014 | Y | Y | Y | Y | Y | Y | Y | Y | NA | 8 |
| 1. Sandhu et al., 2018 | Y | Y | Y | NR | Y | Y | Y | Y | Y | 8 |
| 1. Shongwe et al., 2021 | Y | Y | NA | NR | Y | Y | Y | Y | Y | 7 |
| 1. Siziya et al., 2012 | Y | Y | NA | NR | Y | Y | Y | Y | Y | 7 |
| 1. Sricharan Pasupulati et al., 2013 | Y | Y | Y | Y | Y | Y | Y | Y | Y | 9 |
| 1. Tarafa et al., 2022 | Y | Y | Y | NA | Y | Y | Y | Y | Y | 8 |

**Key:** **Y**= Yes; **NR**= Not reported, **NA**=Not appropriate

**Question codes:**

1. Was the sample frame appropriate to address the target population?

2. Were study participants sampled in an appropriate way?

3. Was the sample size adequate?

4. Were the study subjects and the setting described in detail?

5. Was the data analysis conducted with sufficient coverage of the identified sample?

6. Were valid methods used for the identification of the condition?

7. Was the condition measured in a standard, reliable way for all participants?

8. Was there appropriate statistical analysis?

9. was the response rate adequate, and if not, was the low response rate managed appropriately?
